# Ultrasound-Guided Thermal Ablation of Bethesda IV Thyroid Nodules: A Pilot Study

**DOI:** 10.3389/fendo.2021.674970

**Published:** 2021-08-24

**Authors:** Xinyang Li, Yu Lan, Nan Li, Lin Yan, Jing Xiao, Mingbo Zhang, Yukun Luo

**Affiliations:** ^1^School of Medicine, Nankai University, Tianjin, China; ^2^Department of Ultrasound, The First Medical Center of Chinese People’s Liberation Army (PLA) General Hospital, Beijing, China

**Keywords:** thyroid nodules, Bethesda IV, ultrasound, surgery, thermal ablation

## Abstract

**Objective:**

The purpose of our study was to evaluate the effectiveness of thermal ablation (TA) for Bethesda IV thyroid nodules, and to compare TA and surgery in terms of treatment outcomes, complications, and costs.

**Method:**

This study was approved by the local ethics committee. From January 2017 to December 2019, 30 patients elected TA and 31 patients elected surgery for treatment of Bethesda IV thyroid nodules. Demographics information and conventional ultrasound before treatment for each patient was obtained. For the TA group, the ablation extent was 3 mm beyond the edge of the tumor to prevent marginal residual and recurrence. Patients were followed up at 1, 3, and 6 months after intervention, and every 6 months thereafter. Postoperative complications, operation time, hospitalization time, blood loss, and incision length were recorded.

**Results:**

In the TA group, the volume reduction ratio (VRR) was 94.63 ± 8.99% (range:76%-100%) at the final follow-up. The mean follow-up time was 16.4 ± 5.2months (range:12–24 months). No recurrences, no metastatic lymph node, and no distant metastases were detected during follow-up. The TA group had fewer complications, shorter operation time, smaller incision length, less blood loss, shorter hospitalization time, and lower treatment costs compared to the surgery group (all *P*<0.001).

**Conclusions:**

TA is technically feasible for the complete destruction of Bethesda IV thyroid nodules, and also safe and effective during the follow-up period, with high VRR and low complication rates, especially in patients who were ineligible for or refused surgery.

## Introduction

Thyroid nodule has become increasingly common in clinical practice. This trend possibly stems from the improved detection of asymptomatic and incidental thyroid nodules due to the application of high-resolution ultrasound or other imaging. It has been reported that the incidence of thyroid nodules in adults can be up to 60% (1).

Ultrasound-guided fine-needle aspiration (FNA) is the preferred procedure to evaluate suspicious thyroid nodules, and the Bethesda System for Reporting Thyroid Cytopathology stratifies the malignancy risk based on FNA result. However, the diagnosis of Bethesda IV accounts for 2%-25% (2). The Bethesda IV is an indeterminate cytology type, including follicular neoplasm or suspicion of a follicular neoplasm, which are estimated to have a 10%-40% risk of becoming malignant tumors (3). The indeterminate result creates a management difficulty, and the American Thyroid Association’s guidelines recommend diagnostic lobectomy and molecular testing for the management of follicular neoplasm cytology nodules (2). Lobectomy is the definitive diagnosis tool to distinguish malignant tumors from adenoma, which is based on the presence of vascular or capsular invasion; molecular testing may be used to supplement risk assessment (4). With the increased incidence of Bethesda IV thyroid nodules, an increased number of elderly patients, and the fact that most of such nodules are often benign, more patients are either high surgical risk and ineligible to undergo surgery or unable to afford the high cost of molecular testing; they may also refuse surgery, hoping for other more conservative treatments.

TA, such as radiofrequency ablation (RFA) or microwave ablation (MWA), has been validated for its efficacy and safety in treating thyroid benign nodules and autonomously functioning thyroid nodules (5–7). Recently, TA has also achieved favorable results for the treatment of papillary thyroid carcinomas (PTC) and locally recurrent thyroid cancers (8, 9), and has even shown good performance in the management of T1bN0M0 PTC (10). However, there is little research to suggest thermal ablation should be applied in the treatment of Bethesda IV thyroid nodules or that has evaluated the effectiveness.

In this study, we analyzed the therapeutic outcomes of TA for 30 Bethesda IV thyroid nodules patients after a mean follow-up of 16.4 ± 5.2months (range 12–24 months). Moreover, we also analyzed 31 Bethesda IV thyroid nodules patients who had undergone surgery. Two methods in terms of technical effectiveness, treatment outcomes, complications, and costs were evaluated.

## Materials and Methods

### Patients

**Figure 1:**
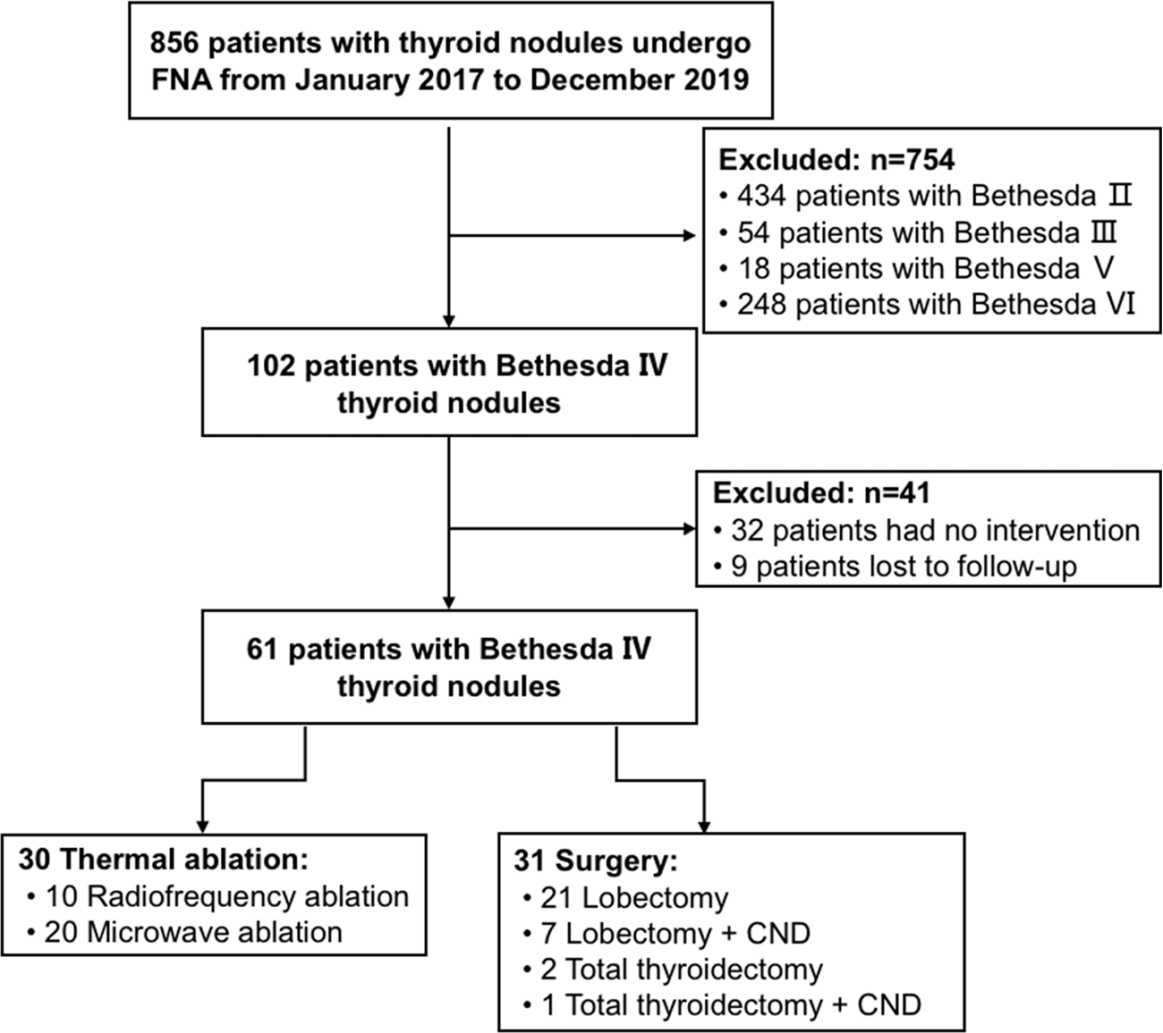
Flowchart summarizing the patient inclusion process. CND is the abbreviation of central lymph node dissection.

This study was approved by the ethics committee of the Chinese People’s Liberation Army General Hospital (S2019-211-01). Written informed consent was obtained from all patients prior to ultrasound-guided FNA, TA, and surgery. We reviewed the medical records of 61 patients who were diagnosed as Bethesda IV thyroid nodules by FNA in our department from January 2017 to December 2019, including 30 patients of ultrasound-guided TA treatment and 31 patients of surgical treatment (**Figure 1**).

For the TA group, patients who fulfilled the following criteria were enrolled: (1) a solitary suspicious thyroid nodule was detected by ultrasound; (2) located in the thyroid gland and with no thyroid capsule contact; (3) no imaging evidence of extrathyroidal invasion, lymph node metastasis, or distant metastasis; (4) ultrasound-guided FNA confirming Bethesda IV cytology; (5) refusal of or ineligibility for surgery due to medical contraindications or other reasons; and (6) complete records and more than 12 months of follow-up.

For the surgery group, the inclusion criteria were as follows: (1) a solitary suspicious thyroid nodule was detected by ultrasound; (2) no imaging evidence of extrathyroidal invasion, lymph node metastasis, or distant metastasis; (3) ultrasound-guided FNA confirming Bethesda IV cytology; and (4) complete clinical data.

For the TA group and the surgery group, the exclusion criteria were: (1) suspicious cervical lymph node metastasis, (2) a history of head or neck irradiation, (3) severe diseases that cannot tolerate interventional and surgical treatment, and (4) a history of severe bleeding and coagulopathy.

### Preoperative Evaluation

Demographics information for each patient was obtained, and all patients underwent conventional ultrasound before treatment to evaluate nodule characteristics, including the diameters in three dimensions (transverse, longitudinal, and anteroposterior), volume, position, composition, echogenicity, shape, margin, and echogenic foci. The volume of each nodule was calculated as V =πabc/6 (where V is the volume; a, b and c are three diameters respectively). For all patients, Bethesda IV thyroid nodules was diagnosed based on cytology findings by ultrasound-guided FNA.

### Ablation Procedure

The ablation techniques include RFA and MWA, which are selected according to nodule characteristics, the relationship with adjacent organs, the patient’s own condition, and the physician’s experience. All TA procedures were performed by one physician with rich experience in thyroid ultrasound and interventional ultrasound, including initial diagnosis and preoperative evaluation. TA was performed in an outpatient ultrasound interventional room. Patients were placed in the supine position. Ultrasound was used to guide the positioning, and then conventional sterilization and sterile drapes were carried out. 1% lidocaine was used for local anesthesia. In order to protect normal structures, using the Hydrodissection technique, the tumor was separated from the critical structures by infusing a mixture of normal saline (11, 12). Before ablation, the approach route and the relationship between the tumor and vital cervical structures (i.e., the trachea, esophagus, recurrent laryngeal nerve, and blood vessels) should be evaluated. During ablation, ultrasound imaging was used for real-time monitoring to ascertain the correct position of the needle in the tumor.

### RFA

The Siemens Acouson Sequoia 512 (Siemens, Mountain View, CA) scanner with a 6.0 MHz linear array transducer was used for guidance. The bipolar RFA generator (CelonLabPOWER; Olympus Surgical Technologies Europe, Hamburg, Germany) and a 18-gauge bipolar RF applicator with a 1.5cm active tip (CelonProSurge micro 100-T15; Olympus Surgical Technologies Europe) were used. Under the guidance of ultrasound, the electrode was inserted into the nodule. Ablation started at 3W of power, and the moving-shot technique was applied (13, 14). If there is no hyperechoic zone at the electrode tip within 5-10 seconds, the RF power was gradually increased. The procedure was not terminated until the whole tumor transformed into a transient hyperechoic zone, and the needle track was coagulated to prevent tumor cell seeding. The whole nodule with a 3mm safety margin was completely ablated to prevent marginal recurrence (15). If the tumor was close to thyroid capsule and less than 3mm, we ablated it together with capsule. At the end of each procedure, the ablation zone was evaluated by contrast-enhanced ultrasound; if residual enhancement was present, ablation was supplemented. All of the patients were discharged when their vital signs were stable after observation for 2 hours.

### MWA

An MTI-5DT machine (Changcheng Microwave System Engineering Co., Ltd., Nanjing, Jiangsu Province, China) and XR-B1610W superficial organ ablation needle (17G, length: 100mm, diameter: 3mm) were used. When the ablation position was determined, the skin was incised 3mm subcutaneously, and the 17G-100 microwave needle was applied by moving-shot technique. After MWA was started the initial power was set to 25W. If there was no hyperechoic zone in the ablative area after 5-10s ablation with this power, the power was gradually increased (up to 35 W). Multifaceted and multipoint mobile ablation was performed until the lesion nodules were completely covered by hyperechogenic vaporization area. The ablation extent was 3 mm beyond the edge of the tumor to prevent marginal residual and recurrence. Contrast-enhanced ultrasound was performed immediately after ablation to evaluate the ablation area. If the ablation was incomplete, the ablation was supplemented; otherwise, the ablation was completed. After observation for 2 hours, patients were discharged with stable vital signs.

### Surgery

Surgery was performed by surgeons with rich experience in thyroid surgery. The decision to perform different surgical methods was made by individual surgeons and patients based on patient preferences in consultation with the surgeon.

After anesthesia was satisfied, the routine neck and shoulder surgery area was disinfected. An arc incision was taken to cut skin, subcutaneous tissue, and latissimus muscle. The thyroid gland was explored, the character of the nodule and the adjacent tissue was evaluated, and the resection was performed. The blood vessels were carefully separated and ligated. During the operation, the recurrent laryngeal nerve and parathyroid gland was protected. The tissue was cut and sent to frozen examination. Neurological function and bleeding were evaluated after surgery.

### Postoperative Evaluation

For the TA group, patients were followed up at 1, 3, 6, and 12 months after intervention, and every 6 months thereafter. The therapeutic effect was evaluated by ultrasound and volume of ablation was detected. The percentage reduction of the ablation area in volume was calculated as follows: Volume reduction ratio (VRR) = [(initial volume – final volume) × 100]/initial volume. Postoperative complications (voice hoarseness, hematoma, postoperative pain, incision infections, hypothyroidism, and hypocalcemia) (16), operation time, hospitalization time, blood loss, and incision length were recorded. Imaging was used to rule out the occurrence of distant metastasis and lymph node metastasis.

### Statistical Analysis

Using SPSS 23.0 software, continuous variables were expressed as mean ± SD, and t-test was used for comparison between groups; categorical data are presented as frequencies and percentages, and Pearson x^2^ test or Fisher exact test was used for comparison between groups. Wilcoxon’s signed rank test was used to compare changes in the maximum diameter and volume of ablation before TA and at each follow-up point. *P* < 0.05 was considered statistically significant.

## Results

### Clinical Characteristics

61 patients with Bethesda IV thyroid nodules were evaluated, including 30 patients of ultrasound-guided TA treatment and 31 patients of surgical treatment. The general data are provided in **Table 1**. In the TA group, there were four males (13.33%) and 26 females (86.67%) aged between 18-68 years (mean 47.3 ± 13.6 years). The surgery group included eight (25.81%) males and 23 (74.19%) females aged between 23–67 years (mean: 48.9 ± 12.5 years). There were no differences between the two groups, including age and sex (*P* > 0.05). Ultrasound characteristics of all the nodules are shown in **Table 1**. For the TA group, the nodule maximum diameter and nodule volume were 2.46 ± 1.37 cm and 6.56 ± 9.09 ml. For the surgery group, the nodule maximum diameter and nodule volume were 2.47 ± 1.35 cm and 6.42 ± 7.10 ml. There were no significant differences between the two groups (*P* > 0.05). The ultrasound characteristics of all thyroid nodules including position, composition, echogenicity, shape, margin, and echogenic foci also showed no significant differences between the two groups (*P* > 0.05).

**Table 1:** General information and ultrasound characteristics of patients undergoing thermal ablation and surgery.

Characteristics	Thermal ablation	Surgery	*P* value
Age(y)	47.3 ± 13.6	48.2 ± 11.5	0.798
Sex			0.221
Male	4	8	–
Female	26	23	–
Nodule maximum diameter(cm)	2.46 ± 1.37	2.47 ± 1.35	0.975
Nodule volume(ml)	6.56 ± 9.09	6.42 ± 7.10	0.945
Position			0.353
Left	11	13	–
Right	19	16	–
Isthmus	0	2	–
Composition			0.949
Cystic or almost completely cystic	0	0	–
Mixed cystic/solid	6	6	–
Solid or almost completely solid	24	25	–
Echogenicity			0.466
Hypoechoic	14	19	–
Isoechoic	14	9	–
Hyperechoic	2	3	–
Shape			0.981
Wider-than-tall	29	30	–
Taller-than-wide	1	1	–
Margin			0.724
Smooth	19	17	–
Ill-defined	8	11	–
Lobulated or irregular	3	2	–
Echogenic foci			0.816
None or large comet-tail artifacts	22	21	–
Macrocalcification	0	0	–
Peripheral calcifications	1	2	–
Punctuate echogenic foci	7	8	–

Continuous variables were expressed as mean ± standard deviation; categorical data are presented as frequencies.

### Follow-Up of TA Patients

**Figure 2:**
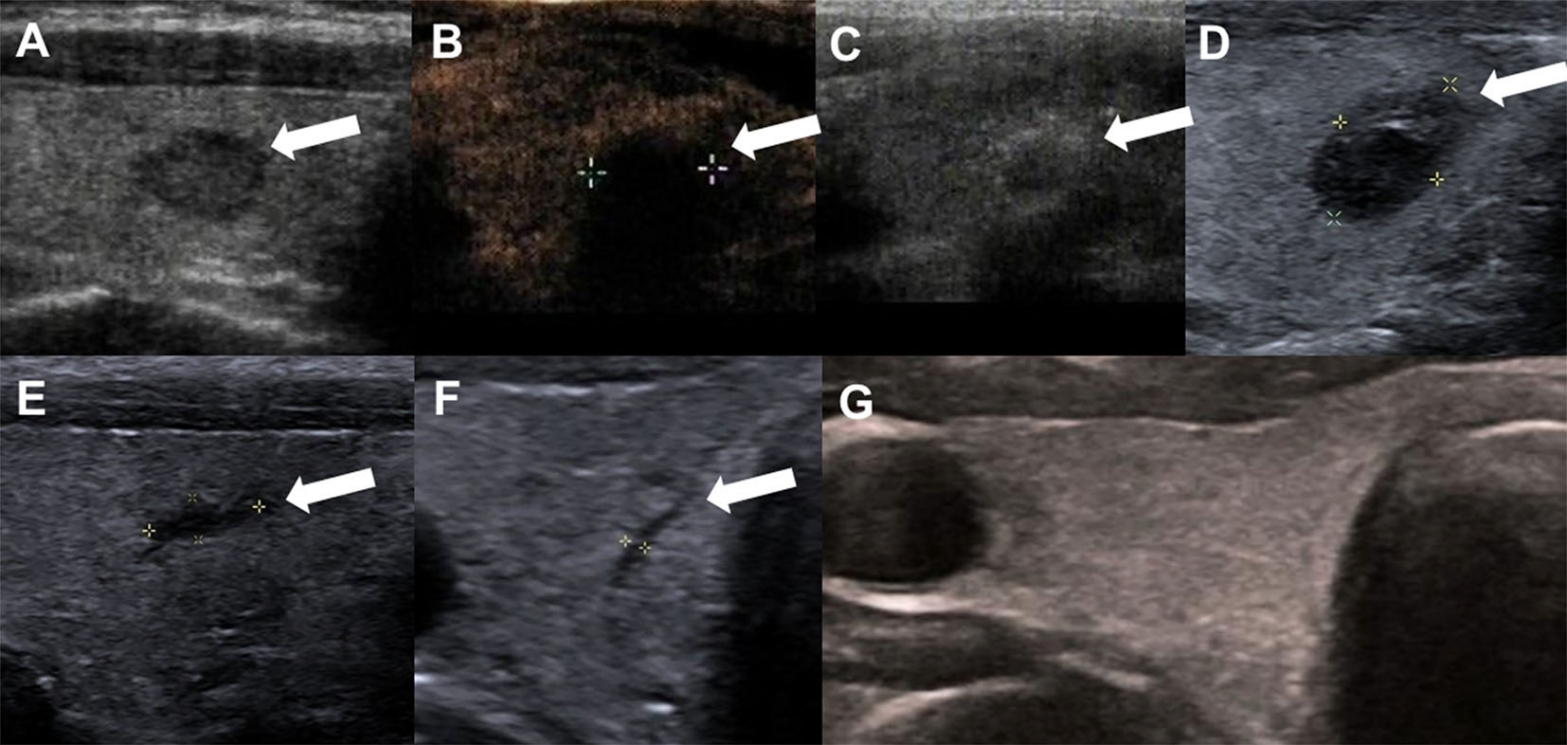
A 47-year-old woman with Bethesda IV thyroid nodules on FNA in the right thyroid lobe. **(A)** Before RFA, ultrasound image shows a hypoechoic nodule with an smooth margin thyroid nodule, and size with 0.9cm×0.6 cm×0.6 cm; **(B)** Contrast-enhanced ultrasound performed immediately after RFA shows larger in size(1.2cm×1.1cm×1.1 cm) than that before ablation and a complete lack of enhancement in the treated area (white arrows). **(C)** Ultrasound shows gas formation in the nodule which covered by a hyperechoic ablation area (white arrows); **(D)** 1 month after ablation, the ablation zone was 0.9cm × 0.5cm × 0.6 cm in size; **(E)** 3 month after RFA, ultrasound shows the treated area shrunk (0.6cm×0.2cm×0.2cm); **(F)** 6 months after RFA, ultrasound shows line-like hypoechoic area, remained as a small scar lesion (white arrows); **(G)** The ablation area disappeared on ultrasonography 12 months after ablation.

**Figure 3:**
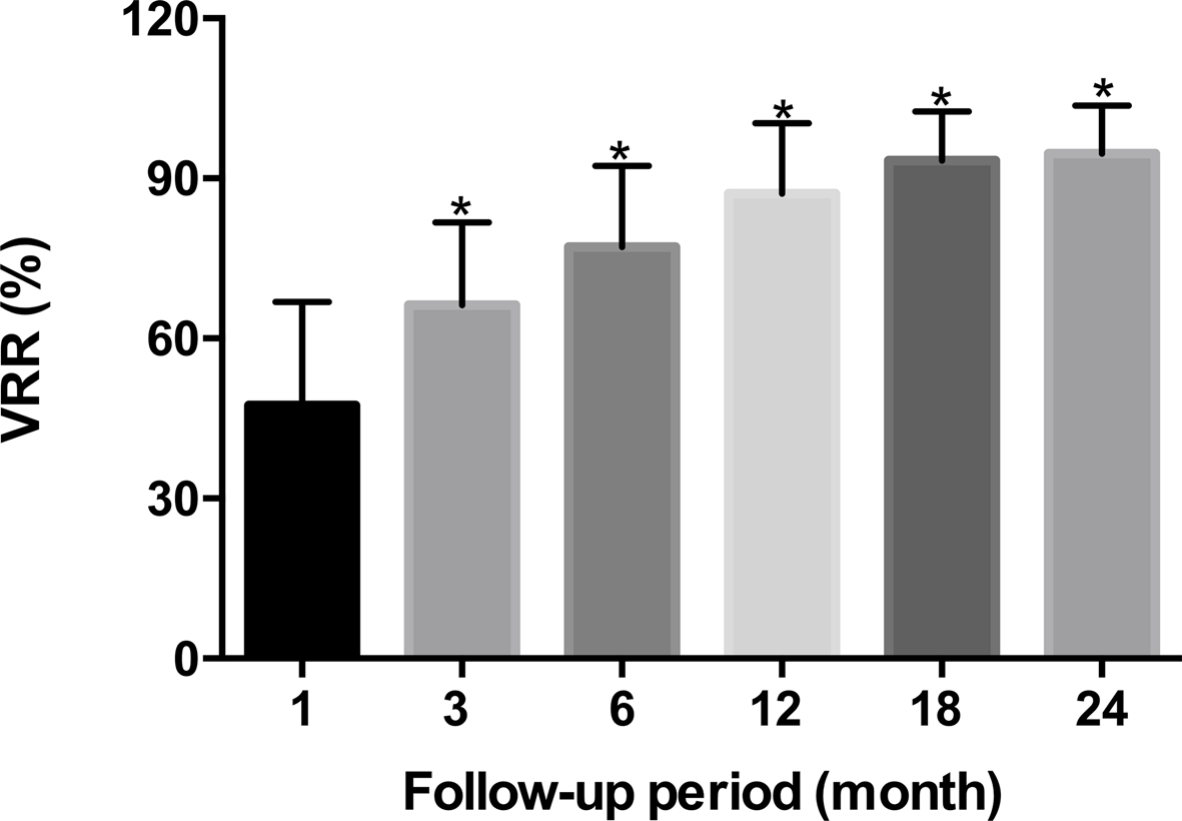
The variation of VRR after thermal ablation at each follow-up period. *p < 0.001.

The 30 patients undergoing TA were performed in accordance with the surgical plan. Immediate contrast-enhanced ultrasound showed that the ablation area was not enhanced, as the maximum diameter and volume was larger than the tumor, which then decreased gradually during the mean 16.4 ± 5.2months follow-up period (**Table 2**, **Figure 2**). There were significant differences in the maximum diameters between baseline and each follow-up point (*P <*0.001), except for immediately and 3 months after ablation (*P* > 0.05) (**Table 2**). The tumor volume showed significant differences between the volume at baseline and that at each follow-up point (*P <*0.001) (**Table 2**). The VRR gradually increased with the extension of follow-up time, and significant differences in the VRR were found at each follow-up point before 24 months (*P <*0.001) (**Table 2**, **Figure 3**). No recurrences, no metastatic lymph node, and no distant metastases were detected during the follow-up.

**Table 2:** Changes of the maximum diameter, mean volume, and reduction rate of the nodule after thermal ablation at each follow-up point.

Follow-up	n	Maximum diameter			Volume			VRR		
		Mean ± SD(cm)	Range	*P* value	Mean ± SD(ml)	Range	*P* value	Mean ± SD(%)	Range	*P* value
Baseline	30	2.46 ± 1.37	0.70-5.20	–	6.56 ± 9.09	0.08-37.90	–	–	–	–
Immediately	30	2.76 ± 1.01	1.20-4.90	0.314	8.45 ± 8.22	0.76-32.00	0.043*	–	–	–
1 month	30	2.37 ± 0.88	0.90-4.00	0.143	4.58 ± 5.10	0.14-22.08	0.019*	47.40 ± 19.47	10-83	<0.001*
3 months	30	2.00 ± 0.82	0.60-4.00	0.003*	2.62 ± 2.70	0.01-11.00	<0.001*	66.27 ± 15.47	34-93	<0.001*
6 months	30	1.71 ± 0.85	0.00-3.80	<0.001*	1.93 ± 2.24	0.00-9.15	<0.001*	77.13 ± 15.25	40-100	<0.001*
12 months	30	1.27 ± 0.91	0.00-3.70	<0.001*	1.34 ± 2.02	0.00-8.54	<0.001*	87.17 ± 13.14	56-100	<0.001*
18 months	14	0.88 ± 1.10	0.00-3.50	<0.001*	0.91 ± 1.71	0.00-6.26	0.002*	93.36 ± 9.17	70-100	<0.001*
24 months	8	0.71 ± 1.22	0.00-3.20	<0.001*	0.79 ± 1.62	0.00-4.52	0.014*	94.63 ± 8.99	76-100	<0.001*

Data are means ± standard deviation.

*P < 0.05 was considered statistically significant.

### Surgery

All patients successfully underwent surgery under general anesthesia, and the histological evaluation of surgical specimens was conducted by experienced pathologists from our department. The pathological results of 31 patients included follicular adenomas (16 cases, 51.61%), Hürthle cell adenomas (2 cases, 6.45%), nodular goiters (2 cases, 6.45%), follicular thyroid carcinoma (FTC) (3 cases, 9.68%) [1 of T1bN0M0 and 2 of T2N0M0, according to The American Joint Committee on Cancer TNM system for differentiated thyroid cancer (17)] including 2 minimally invasive carcinomas, non-invasive follicular thyroid neoplasm with papillary-like nuclear features (1 case, 3.23%), and follicular tumor of uncertain malignant potential (7 cases, 22.58%).

### Complications and Procedures Condition

Of the 30 patients in the TA group, only one patient had postoperative pain after ablation, which resolved spontaneously in 2 hours. For the surgery group, 31 patients had postoperative pain. Thyroid function was assessed 1 month after surgery. Four patients were diagnosed with hypothyroidism and received prescriptions for levothyroxine sodium, and one patient had subclinical hypothyroidism. Parathyroid function was assessed 1 day and 1 month after surgery. Four patients had transient hypoparathyroidism, three of which were asymptomatic; the remaining patient had mild facial paresthesia. The serum parathyroid hormone and calcium level of all the four patients were restored within 3 months after calcium supplement treatment. Therefore, a significant difference was noted (**Table 3**) when compared with the TA group. The TA group had a shorter operation time, smaller incision length, less blood loss, shorter hospitalization time, and lower treatment costs compared to the surgery group (all *P <*0.001, **Table 4**).

**Table 3:** Comparison of the complications of the thermal ablation group and surgery group.

Comparison	Thermal ablation	Surgery	*P* value
Voice hoarseness	0	0	–
Hematoma	0	0	–
Postoperative pain	1	31	<0.001*
Incision infections	0	0	–
Hypothyroidism	0	5	<0.001*
Hypocalcemia	0	4	0.042*

Data are numbers of patients.

*P < 0.05 was considered statistically significant.

**Table 4:** Treatment variables of the thermal ablation and the surgery groups.

Characteristics	Thermal ablation (n=30)	Total Surgery (n=31)	*P* valve
Total operation time(min)	4.43 ± 2.36	85.55 ± 28.29	<0.001*
Estimated Blood loss(ml)	0	36.93 ± 51.03	<0.001*
Hospitalization(d)	0	6.84 ± 1.72	<0.001*
Cost($USD)	1900.62 ± 119.58	2932.89 ± 529.70	<0.001*
Incision length(cm)	0.19 ± 0.15	6.52 ± 1.36	<0.001*

Data are means ± standard deviation.

*P < 0.05 was considered statistically significant.

## Discussion

After more than 20 years of development, ultrasound-guided TA has been widely used as a minimally invasive technique for the management of liver, kidney, lung, and bone tumors (18–21). Recently, some studies have been shown the efficacy and safety of TA in the treatment of clinically relevant benign thyroid nodules (5, 22, 23), localized small recurrent thyroid cancers (9), and low risk PTC (8, 10, 24). In our study, 30 patients with Bethesda IV thyroid nodules were treated with TA. These patients were ineligible for or refused surgery. We demonstrated that TA might be safe, effective, and have low complication rates for the treatment of Bethesda IV patients during the mean 16.4 ± 5.2months follow-up period.

Ultrasound-guided FNA and The Bethesda System for Reporting Thyroid Cytopathology can stratify the thyroid nodules, which is an essential diagnostic tool that is used to help to understand the nature of thyroid nodules and assist clinicians in selecting the management scheme for patients (2, 3). However, indeterminate cytology results account for 20% of biopsied nodules, posing a significant management dilemma (25). And diagnostic lobectomy and molecular testing are recommended according to the present American Thyroid Association’s management guidelines (2). But frozen section pathology at the time of surgery cannot offer reliable pathological results, and instead must rely on the postoperative permanent histologic sections. FTC generally must undergo a repeat surgery, which is difficult and full of challenges for patients and surgeons. Molecular testing may be useful to determine the status of follicular lesions as more or less likely to be malignant based on the genetic profile, but it needs further validation and utility studies and its cost-effectiveness should also be considered (25, 26). In our study, the final pathological results of 31 patients who underwent surgery with Bethesda IV thyroid nodules are follicular adenomas, Hürthle cell adenomas, nodular goiters, FTC, non-invasive follicular thyroid neoplasm with papillary-like nuclear features, and follicular tumor of uncertain malignant potential. Such nodules are often benign but may also be potentially aggressively malignant. In our surgery group, there are three patients with FTC (9.68%), two of whom have minimally invasive carcinomas. FTC is a differentiated malignant tumor like PTC (27). Differentiated thyroid carcinomas usually have an excellent prognosis, exceeding 90% to 95% of 10-year survival rates (28). For comparable age and disease stage, the prognoses of PTC and FTC are similar (29, 30). But FTC may be more vascularly invasive and clinically aggressive than PTC (31). NIFTP has a low risk for adverse outcomes and requires less aggressive treatment (32). Minimally invasive follicular carcinomas are less likely to produce distant metastases, similar to follicular adenomas (33). At present, TA has also achieved favorable results for the treatment of PTC and recurrent thyroid cancers (8, 9), and even for T1bN0M0 PTC (10). For the TA group, the ablation extent was 3 mm beyond the edge of the tumor to prevent marginal residual and recurrence. In addition, some studies have shown that TA mainly destroys tumor tissue through physical effects, and tumor debris releasing tumor antigens *in situ* may provoke systemic anti-cancer immunity, so this is analogous to endogenous vaccination. This would also affect and eliminate hidden metastatic cancer (34). In our study, no recurrences, lymph node metastasis, or distant metastasis were detected in 30 patients with Bethesda IV thyroid nodules during the 24 months follow-up period. A longer follow-up period is needed to focus on the occurrence of the metastasis and recurrences (35). Ha SM et al. (36) also showed 10 follicular neoplasms of TA with no recurrences or distant metastases during the 5 years follow-up period. Dobrinja et al. (37) showed two patients with follicular neoplasms who underwent a total thyroidectomy after RFA, and confirmed that the procedure did not affect subsequent thyroid surgery and histological diagnosis. But the small sample size cannot clarify the effectiveness to the ablation treatment of follicular neoplasms. Therefore, for patients who were ineligible for or refused surgery, TA may be a viable option. We should choose patients carefully, and then use the advantages of TA to destroy Bethesda IV thyroid nodules and prevent marginal residual and recurrence.

In our study, we reviewed 30 Bethesda IV patients who underwent TA. The VRR was 94.63 ± 8.99% (range:76%-100%) at 24 months. According to this standard, effective treatment was defined as a VRR > 50% of the initial nodule volume (38). The therapeutic success rate of our study was 100%. 7 Bethesda IV nodules had completely disappeared during the follow-up time, and 19 Bethesda IV nodules had a greater VRR than 90%. Some retrospective studies describe a 67–96% mean volume reduction after 12 months (39–42), which is in line with the findings of our study. This wide range may be attributed to the difference in age, size, and structure of thyroid nodules, and currently available TA techniques. Our results suggest that TA is an effective and safe procedure for patients with Bethesda IV thyroid nodules, and for those who are ineligible for or refused surgery.

In terms of complications, our study shows that TA was less invasive than surgery. In the TA group, only one patient had postoperative pain which resolved spontaneously. In the surgery group, in addition to postoperative pain, there were also complications of hypothyroidism and hypocalcemia, which needed drug supplement treatment. TA offers better protection of thyroid function. The TA group had a shorter intervention time, smaller incision length, less blood loss, shorter hospitalization time, and lower treatment costs compared to the surgery group. All patients of the TA group were treated in one session. If repeated TA for recurrence of nodule is required, the cost would be higher than surgery. However, this risk is low and requires longer follow-up to verify (35). Therefore, TA is a cost-effective and risk-effective technique, which can not only destroy nodules and relieve local symptoms, but also is less invasive.

There were many limitations in our study. The number of patients was small, and follow-up time was not long enough. Validation of the results requires a large population, a long follow-up period, and prospective and multicenter studies. For the TA group, the final pathology of Bethesda IV thyroid nodules has not been diagnosed. In the future, development in the field of molecular testing and emergence of new technologies are needed to assist in the differential diagnosis between follicular adenomas and FTC.

In conclusion, this study demonstrated ultrasound-guided TA is technically feasible for the complete destruction of Bethesda IV thyroid nodules, and is also safe and effective during mean 16.4 ± 5.2months follow-up period, with high tumor VRR and low complication rates. Surgical treatment is the first choice, but for patients who are ineligible for or refuse surgery, TA may be an available option. The persistence and repeatability of these findings in our study need to be demonstrated by larger populations and longer follow-up periods.

## Data Availability Statement

The raw data supporting the conclusions of this article will be made available by the authors, without undue reservation.

## Ethics Statement

The studies involving human participants were reviewed and approved by institutional review board of General Hospital of Chinese PLA(S2019-211-01). The patients/participants provided their written informed consent to participate in this study.

## Author Contributions

XL and YKL: integrity of the whole study, analysis of data, and review of the final manuscript. MZ and YL: management of data and manuscript writing. LY, JX, and NL: statistical analysis of data. All authors contributed to the article and approved the submitted version.

## Funding

This work was supported by the National Natural Science Foundation of China under Grant [No. 81771834].

## Conflict of Interest

The authors declare that the research was conducted in the absence of any commercial or financial relationships that could be construed as a potential conflict of interest.

## Publisher’s Note

All claims expressed in this article are solely those of the authors and do not necessarily represent those of their affiliated organizations, or those of the publisher, the editors and the reviewers. Any product that may be evaluated in this article, or claim that may be made by its manufacturer, is not guaranteed or endorsed by the publisher.
